# RNA recognition by minimal ProQ from *Neisseria meningitidis*

**DOI:** 10.1261/rna.080207.124

**Published:** 2025-04

**Authors:** Maciej Basczok, Mikołaj Olejniczak

**Affiliations:** Institute of Molecular Biology and Biotechnology, Faculty of Biology, Adam Mickiewicz University, 61-614 Poznań, Poland

**Keywords:** *Neisseria* ProQ, the FinO domain, bacterial regulatory RNA, RNA-binding proteins in bacteria

## Abstract

*Neisseria meningitidis* minimal ProQ is a global RNA-binding protein belonging to the family of FinO-domain proteins. The *N. meningitidis* ProQ consists only of the FinO domain accompanied by short N- and C-terminal extensions. To better understand how this minimal FinO-domain protein recognizes RNAs, we compared its binding to seven different natural RNA ligands of this protein. Next, two of these RNAs, *rpmG*-3′ and AniS, were subject to further mutational studies. The data showed that *N. meningitidis* ProQ binds the lower part of the intrinsic transcription terminator hairpin, and that the single-stranded sequences on the 5′ and 3′ side of the terminator stem are required for tight binding. However, the specific lengths of 5′ and 3′ RNA sequences required for optimal binding differed between the two RNAs. Additionally, our data show that the 2′-OH and 3′-OH groups of the 3′ terminal ribose contribute to RNA binding by *N. meningitidis* ProQ. In summary, the minimal ProQ protein from *N. meningitidis* has generally similar requirements for RNA binding as the isolated FinO domains of other proteins of this family, but differs from them in detailed RNA features that are optimal for specific RNA recognition.

## INTRODUCTION

FinO-domain proteins are a diverse family of RNA-binding proteins, which are present in many proteobacteria (Glover et al. 2015; Attaiech et al. 2017; Olejniczak and Storz 2017; Holmqvist and Vogel 2018; Holmqvist et al. 2020). Besides the RNA-binding FinO domain, these proteins also often contain N-terminal or C-terminal extensions, which contribute to their physiological functions (Arthur et al. 2003; Attaiech et al. 2016; El Mouali et al. 2021b; Rizvanovic et al. 2021). The FinO-domain proteins bind to regulatory RNAs and mRNAs (van Biesen and Frost 1994; Attaiech et al. 2016; Smirnov et al. 2016; Holmqvist et al. 2018; Melamed et al. 2020), and contribute to the regulation of important physiological processes, including the F plasmid transfer (van Biesen and Frost 1992; Glover et al. 2015), natural transformation (Attaiech et al. 2016), adaptation to available nutrients (El Mouali et al. 2021b), motility (Rizvanovic et al. 2021), and bacterial virulence (Westermann et al. 2019; Rizvanovic et al. 2022; Bergman et al. 2024). In bacterial cells, FinO-domain proteins coexist with a matchmaker protein Hfq (Updegrove et al. 2016; Kavita et al. 2022; Malecka and Woodson 2024), but ProQ and Hfq mostly recognize different RNA targets (Holmqvist et al. 2018; Melamed et al. 2020). Although all FinO-domain proteins have the same RNA-binding domain, there are wide differences between them in RNA recognition, because some bind few RNAs (van Biesen and Frost 1994; Attaiech et al. 2016; Gerovac et al. 2020; El Mouali et al. 2021a), while others are global RNA binders (Holmqvist et al. 2018; Bauriedl et al. 2020; Melamed et al. 2020).

Intrinsic transcription terminators are the binding sites of FinO-domain proteins in small RNAs and mRNAs (Arthur et al. 2011; Attaiech et al. 2016; Holmqvist et al. 2018; Bauriedl et al. 2020; Melamed et al. 2020; Stein et al. 2020; Kim et al. 2022). The FinO domain forms a compact shape with clearly defined convex and concave surfaces (Ghetu et al. 2000; Chaulk et al. 2010; Gonzalez et al. 2017; Immer et al. 2020; Kim et al. 2022). The concave face has been shown as the RNA-binding site in F-like plasmid FinO (Ghetu et al. 2002), in *Escherichia coli* ProQ (Pandey et al. 2020; Stein et al. 2023), and in *Legionella pneumophila* RocC proteins (Kim et al. 2022). The crystal structure of the isolated FinO domain of *L. pneumophila* RocC with the terminator of RocR sRNA showed how the terminator hairpin with the 3′ tail binds to the concave face of the FinO domain (Kim et al. 2022). Particularly important for the interaction are two regions of the protein. One of them is a group of amino acids in α-helix 5, which side chains contact the phosphor-sugar backbone of the lower part of the terminator stem. The other region is a pocket on the concave face where side chains of conserved tyrosine and arginine together with other residues contact two terminal nucleotides of the 3′ tail of RocR (Kim et al. 2022).

Besides the FinO domain, other regions can also contribute to RNA binding, but such regions are not present in all proteins from this family (Attaiech et al. 2017; Olejniczak and Storz 2017). The N-terminal extension of the F-like plasmid FinO protein contributes to RNA binding and strand exchange (Ghetu et al. 2002; Arthur et al. 2003). Additionally, it was observed that while the isolated FinO domain of *E. coli* ProQ protein can bind only to RNAs containing intrinsic transcription terminators or similar structures on their 3′ ends, the full-length ProQ, which contains a positively charged linker, can bind well also to RNAs devoid of such structures (Stein et al. 2020). The role of the ProQ linker in RNA binding was also proposed using the hydrogen-deuterium exchange studies (Gonzalez et al. 2017). These data suggest that N- or C-terminal extensions can contribute to RNA binding by FinO-domain proteins. However, not all proteins from this family contain such additional regions, which raises the question whether such minimal proteins consisting only of FinO domains recognize RNAs in the same way as the FinO domains of proteins containing large extensions. Two such minimal proteins are *L. pneumophila* Lpp1663, which structure was solved by NMR (Immer et al. 2020), and *Neisseria meningitidis* minimal ProQ (NMB 1681), which structure was solved by X-ray crystallography (Chaulk et al. 2010). While the natural RNA ligands of Lpp1663 are not yet known, those of *N. meningitidis* minimal ProQ have been recently identified using the CLIP-seq method (Bauriedl et al. 2020).

The 141-aa long *N. meningitidis* minimal ProQ protein (NMB 1681) consists mainly of the core FinO domain, and additionally has only short 19-aa long N-terminal, and 13-aa long C-terminal extensions (Chaulk et al. 2010; Olejniczak and Storz 2017). The comparison of the structures of the six copies of the protein present in the crystallographic asymmetric unit showed that the 19-aa long N-terminal extension is likely flexible (Chaulk et al. 2010). Interestingly, while in the FinO domains of F-like plasmid FinO and *E. coli* ProQ, there is a larger positively charged surface on the concave than on the convex face of the domain, in *N. meningitidis* minimal ProQ, it extends on both the concave and the convex face (Chaulk et al. 2010; Olejniczak and Storz 2017).

Recent CLIP-seq study identified almost 200 mRNAs and sRNAs associated with *N. meningitidis* ProQ, in which the ProQ binding sites often overlapped intrinsic transcription terminators (Bauriedl et al. 2020). The direct binding of several of these RNAs to *N. meningitidis* ProQ was further supported by binding assays using purified components (Bauriedl et al. 2020). It was also previously shown that *N. meningitidis* ProQ bound tightly to the transcription terminator derived from FinP RNA (Chaulk et al. 2010).

To elucidate how the *N. meningitidis* minimal ProQ recognizes RNAs, we compared the strength of ProQ binding to several of its natural RNA ligands using gel-shift assay. Next, we used mutant RNAs to determine what RNA features are essential for tight binding to *N. meningitidis* minimal ProQ. The data showed that the bottom part of the Rho-independent transcription terminator hairpin together with adjacent single-stranded sequences are recognized by the minimal ProQ, and that the 3′ terminal ribose has an important role in the interaction. The results of our studies suggest that although the minimal ProQ from *N. meningitidis* recognizes the same general RNA features as other FinO-domain proteins, there are also subtle differences in RNA properties, which are optimal for binding by *N. meningitidis* ProQ in comparison with the isolated FinO domains from other proteins.

## RESULTS

To identify features of the binding sites of *N. meningitidis* ProQ in its RNA ligands, we compared the secondary structures of the regions covered by CLIP-seq peaks in a previously published study (Supplemental Fig. S1; Bauriedl et al. 2020). The results of this analysis showed that in 151 out of 234 previously identified RNA ligands of ProQ, which is ∼65%, the CLIP-seq peak at least partly overlapped with an intrinsic transcription terminator hairpin followed by a U-rich sequence (Supplemental Fig. S1). Intrinsic transcription terminators have already been shown as the binding sites of the FinO domains of *E. coli* and *S. enterica* ProQ, *L. pneumophila* RocC, and F-like plasmid FinO (Arthur et al. 2011; Holmqvist et al. 2018; Melamed et al. 2020; Stein et al. 2020; Kim et al. 2022), and it has also been proposed that they serve as the binding sites of *N. meningitidis* ProQ, which consists solely of a FinO domain (Bauriedl et al. 2020). Among these 151 RNAs, the majority have an A-rich sequence motif consisting of at least two consecutive adenosine nucleotides immediately upstream of the terminator (Supplemental Fig. S1). Among 83 RNA ligands, which do not contain terminator structures are mRNA 5′ UTRs, 3′ UTRs, and intergenic regions, of which more than 30 contain hairpin structures within the ProQ binding sites. Overall, these data suggest that the most common secondary structure motif in the *N. meningitidis* ProQ binding sites within its RNA ligands is an intrinsic transcription terminator hairpin followed by a poly(U) sequence.

**FIGURE 1.**
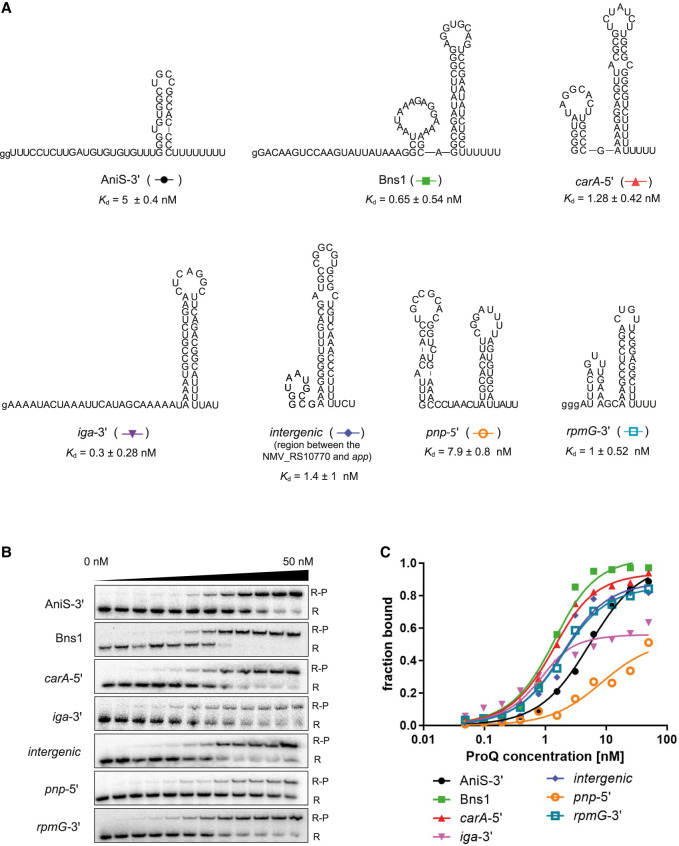
RNA molecules bound by ProQ protein in *N. meningitidis*, which were used in this study. (*A*) The secondary structures of RNAs AniS-3′, Bns1, *carA*-5′, *iga*-3′, *intergenic* (intergenic region between NMV_RS10770::*app*), *pnp*-5′, and *rpmG*-3′. The lower-case g denotes guanosine residue added on 5′ end to enable T7 RNA polymerase transcription. (*B*) The binding of ^32^P-labeled RNAs AniS-3′, Bns1, *carA*-5′, *iga*-3′, *intergenic*, *pnp*-5′, and *rpmG*-3′ to ProQ was monitored using a gel-shift assay. Free ^32^P-RNA is marked as R, RNA-ProQ complexes as R-P. (*C*) The fitting of the ProQ binding data from *B* using the quadratic equation provided *K*_d_ values of 4.9 nM for AniS-3′, 0.9 nM for Bns1, 0.9 nM for *carA*-5′, 0.3 nM for *iga*-3′, 1.4 nM for *intergenic,* 7.9 nM for *pnp*-5′, and 1.2 nM for *rpmG*-3′. The RNA secondary structure predictions were performed in the *ViennaRNA* program (Lorenz et al. 2011). Raw gel data for all RNAs are presented in Supplemental Figure S3. The average equilibrium dissociation constant (*K*_d_) values and the maximum RNA fraction bound calculated from at least three independent experiments are shown in Table 1.

To compare how tightly ProQ binds its different RNA ligands, we selected a set of seven RNAs that represent different classes of RNAs bound by ProQ in *N. meningitidis*, which were previously identified using the CLIP-seq method (Fig. 1A; Bauriedl et al. 2020). In this set were included four RNAs that have already been shown to bind to purified ProQ (Bauriedl et al. 2020), because this provided further support that these are direct ligands of ProQ. The fragments of these seven RNAs used in the study include the whole sequence covered by CLIP-seq peak (Bauriedl et al. 2020), hence, they correspond to ProQ binding sites in these RNAs. Among selected RNA ligands were two sRNAs, Bns1 and AniS, two mRNA 5′ UTRs, *carA*-5′ and *pnp*-5′, two mRNA 3′ UTRs, *rpmG*-3′ and *iga*-3′, and an RNA originating from the intergenic region between the NMV_RS10770 and *app* genes (Fig. 1A). Interestingly, AniS, which was the least enriched among ProQ ligands identified by CLIP-seq (Bauriedl et al. 2020), is also a ligand of *N. meningitidis* Hfq (Fantappie et al. 2011; Heidrich et al. 2017). The in vitro binding of Bns1 sRNA, AniS sRNA, the 5′ UTR of *pnp*, and the 3′ UTR of *rpmG* to purified *N. meningitidis* ProQ have already been shown (Bauriedl et al. 2020). The 3′ ends of sRNAs and mRNA 3′ UTRs used in our binding studies were defined so as they contained the complete terminator structure, which overlapped with the reported CLIP-seq peak (Bauriedl et al. 2020). The 3′ ends of mRNA 5′ UTRs, *carA*-5′ and *pnp*-5′, were defined by the 3′ end of the sequence of the reported CLIP-seq peak (Bauriedl et al. 2020). The 5′ ends were defined to start with guanosine residues present in the natural sequence, or the guanosine residues were added to the 5′ ends, to ensure efficient in vitro transcription. Instead of full-length AniS sRNA, we used its 3′-terminal fragment, including the whole reported CLIP-seq peak (Bauriedl et al. 2020), which we named AniS-3′, and which bound ProQ with similar affinity as full-length AniS (Supplemental Fig. S2). Among these RNAs, four (AniS, Bns1, *iga*-3′, and *rpmG*-3′) contain intrinsic terminator structures, while the other three contain hairpin structures followed by U-rich sequences (Supplemental Fig. S1). In summary, the RNA molecules that we used included 90 nt long Bns1 sRNA, 55 nt long AniS-3′, 65 nt long *pnp*-5′, 64 nt long *carA-*5′, 48 nt long *rpmG*-3′, 64 nt long *iga*-3′, and 55 nt fragment of the intergenic region between the NMV_RS10770 and *app* genes, which we named *intergenic* (Fig. 1A).

We compared the binding affinities of the seven RNAs to ProQ using a gel-shift assay (Fig. 1B,C; Table 1; Supplemental Fig. S3). The data showed that all RNAs formed single complexes with ProQ in the studied concentration range (Fig. 1B; Supplemental Fig. S3). To obtain *K*_d_ values, the binding data were fit to the quadratic equation. The average *K*_d_ values with standard deviation obtained from at least three replicates are presented in Table 1. The binding of all RNAs to ProQ was tight with *K*_d_ values in the low nanomolar range. The binding affinities of these RNAs for ProQ ranged from *K*_d_ value of 0.3 nM for *iga*-3′ to 7.9 nM for *pnp*-5′. However, while the fraction bound of AniS-3′, Bns1, *carA*-5′, *intergenic*, and *rpmG*-3′ reached ∼90% at saturation, that of *iga*-3′ and *pnp*-5′ saturated at only ∼ 50%–60% (Fig. 1B,C; Table 1; Supplemental Fig. S3). This could suggest that these two RNAs formed alternative RNA conformations or that the complexes of these RNAs with ProQ partly dissociated during electrophoresis. As a control, we also calculated the *K*_d_ values using global data fitting (Supplemental Table S1). The *K*_d_ values obtained using both calculation approaches were very close, except for *pnp*-5′ binding to ProQ, which *K*_d_ value was about four times tighter when calculated using the global data fitting. This difference in calculating *K*_d_ value of *pnp*-5′ using the two methods could be a result of the lower maximum fraction of *pnp*-5′ bound, which could affect the accuracy of data fitting.

**TABLE 1.** Equilibrium RNA binding to *N. meningitidis* ProQ

^32^P-RNA	*K*_d_ [nM] (maximal fraction bound)
AniS-3′	5 ± 0.4 (91%)
Bns1	0.65 ± 0.54 (97%)
carA-5′	1.3 ± 0.42 (88%)
iga-3′	0.3 ± 0.28 (51%)
intergenic	1.4 ± 1 (86%)
*pnp*-5′	7.9 ± 0.8 (63%)
rpmG-3′	1 ± 0.52 (89%)

The *K*_d_ values were obtained by fitting the data using the quadratic equation. The average *K*_d_ values with standard deviations were calculated from at least three independent experiments.

Because it was previously observed that longer fragments of *pnp*-5′ and *rpmG*-3′ are also bound by ProQ (Bauriedl et al. 2020), we compared the binding of 3′-extended versions of these two RNAs, which we named *pnp*-5′-ext and *rpmG*-3′-ext (Supplemental Figs. S4, S5). Both *pnp*-5′-ext and *rpmG*-3′-ext RNAs bound ProQ weaker than *pnp*-5′ and *rpmG*-3′, respectively, because the fractions bound of each RNA at the maximum 50 nM concentration used were < 20%. Hence, extending the 3′ end of *rpmG*-3′ beyond the six continuous uridines of the 3′ tail of the terminator, or extending the 3′ end of *pnp*-5′ beyond a terminator-like structure, was detrimental for ProQ binding (Supplemental Figs. S4, S5). This observation is consistent with previous reports that extending RNAs beyond their 3′ poly(U) tails was detrimental for RNA binding by the FinO domain of *E. coli* ProQ (Stein et al. 2020), and that RocR RNA with an elongated 3′ tail bound less well to the FinO domain of *L. pneumophila* RocC protein (Kim et al. 2022).

### 3′ poly(U) tail is required for RNA binding by *N. meningitidis* ProQ

To better understand how *N. meningitidis* ProQ recognizes its RNA ligands, we focused on *rpmG*-3′ and AniS-3′ RNAs. We selected *rpmG*-3′ for further study because it has features typical for many RNA ligands of *N. meningitidis* ProQ. In particular, the ProQ binding site identified in this RNA by CLIP-seq data (Bauriedl et al. 2020) overlaps with an intrinsic transcription terminator, which has an A-rich sequence upstream of the terminator, and is followed by poly(U) tail (Fig. 1A; Supplemental Figs. S1, S6; Supplemental Table S2). Its binding to ProQ has been previously studied (Bauriedl et al. 2020), and in our experiments it bound tightly to ProQ with saturation at ∼ 90% bound (Table 1). While the ProQ binding site in AniS-3′ sRNA also included an intrinsic transcription terminator (Bauriedl et al. 2020), this sRNA has an unusual feature of a U-rich sequence upstream of the terminator (Fig. 1A; Supplemental Fig. S1). The binding of AniS to ProQ has also been previously studied (Bauriedl et al. 2020), and we observed that AniS-3′ has low nanomolar binding affinity to ProQ and its binding also saturated at ∼ 90% (Table 1). Additionally, *rpmG*-3′ and AniS represent two important groups of RNA ligands of ProQ, mRNA 3′ UTRs and sRNAs. We concluded that comparing two RNA ligands of *N. meningitidis* ProQ that have partly different features should allow identifying the sequence and structure determinants of RNA binding to this protein.

**FIGURE 2.**
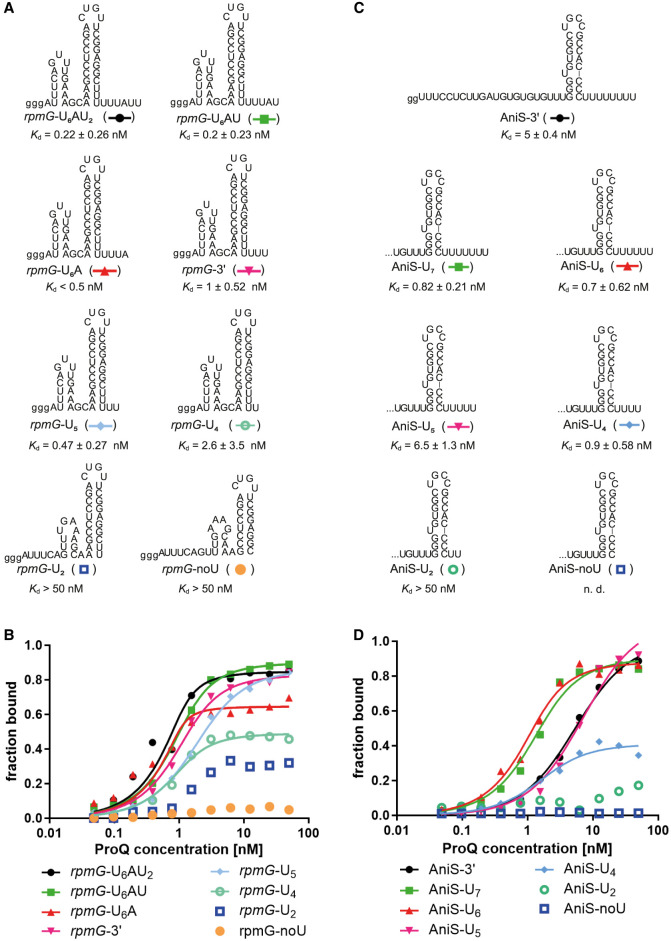
The 3′-terminal poly(U) tail is involved in *rpmG*-3′ and AniS-3′ binding to the *N. meningitidis* ProQ protein. (*A*) *rpmG*-3′ constructs with different lengths of 3′ poly(U) tails. (*B*) The fitting of the ProQ binding data using the quadratic equation provided *K*_d_ values of 0.1 nM for *rpmG*-U_6_AU_2_, 0.4 nM for *rpmG*-U_6_AU, 1.2 nM for *rpmG*-3′, 0.5 nM for *rpmG*-U_5_, and 0.4 nM for *rpmG*-U_4_, while the binding for *rpmG*-U_2_ reached saturation below 40% of bound RNA fraction, and *rpmG*-noU did not reach saturation up to 50 nM concentration of the ProQ. The *K*_d_ value for *rpmG*-U_6_A was estimated as <0.5 nM. (*C*) AniS-3′ constructs with different lengths of 3′ poly(U) tails. (*D*) The fitting of the ProQ binding data using the quadratic equation provided *K*_d_ values of 4.9 nM for AniS-3′, 0.8 nM for AniS-U_7_, 0.5 nM for AniS-U_6_, 6.6 nM for AniS-U_5_, and 1.1 nM for AniS-U_4_, while the binding for AniS-U_2_ did not reach saturation up to 50 nM concentration of the ProQ. The binding of AniS-noU was essentially undetectable up to 50 nM concentration of the ProQ. The data in the plots for *rpmG*-3′ and AniS-3′ binding to ProQ are the same as in Figure 1. The lower case g denotes guanosine residue added on 5′ end to enable T7 RNA polymerase transcription. Gels corresponding to the data in the plots are shown in Supplemental Figure S7. The RNA secondary structure predictions were performed in the *ViennaRNA* program (Lorenz et al. 2011). The average equilibrium dissociation constant (*K*_d_) values and maximum RNA fraction bound calculated from at least three independent experiments are shown in Table 2.

At first, we analyzed how the length of the 3′ terminal poly(U) tail of the terminator affects the binding of *rpmG*-3′ and AniS-3′ RNAs to ProQ (Fig. 2; Supplemental Fig. S7). For *rmpG*-3′, we compared eight variants, which differed with the length of the 3′ tail, including three that were longer than *rmpG*-3′ and included the following sequence of *rpmG* gene, while for AniS-3′, we compared seven length variants, of which AniS-3′ had the longest eight-uridine tail encoded in *aniS* gene. The data showed that the binding of *rpmG*-3′ and AniS-3′ to ProQ differently depended on the 3′ tail length (Fig. 2B,D; Table 2; Supplemental Fig. S7). For *rmpG*-3′, the 3′-tail lengths from 5 to 9 nt ensured tight binding to ProQ. Hence, even the *rmpG*-3′ mutant with the longest 9 nt tail bound tightly to ProQ. On the other hand, the binding of RNA *rmpG*-U_4_ with 3′ tail of 4 nt of length saturated at a maximum fraction bound <60%, that of *rmpG*-U_2_ saturated at <40% fraction bound, and that of *rmpG*-noU was barely detectable (Fig. 2A,B; Table 2; Supplemental Fig. S7A). The data analysis using global fitting also showed that RNAs with 3′ tails longer than that of *rpmG*-3′ had tighter *K*_d_ values than *rpmG*-3′, while the *K*_d_ values of *rmpG*-U_5_ and *rmpG*-U_4_ were similar to those of *rmpG*-3′ (Supplemental Table S1). Overall, these data showed that the binding of RNAs with 3′ tails between 7 and 9 nt, which are longer than that of *rmpG*-3′, resulted in tighter binding to ProQ, those with 3′ tails of 4 and 5 nt bound similarly as *rmpG*-3′, and those with the 3′ tail of 2 nt or devoid of a 3′ tail bound much weaker.

**TABLE 2.** The length of the 3′ single-stranded tail following the Rho-independent terminator affects the binding of *rpmG*-3′ and AniS-3′ RNAs to *N. meningitidis* ProQ.

^32^P-RNA	*K*_d_ [nM] (maximal fraction bound)
*rpmG*-U_6_AU_2_	0.22 ± 0.26 (88%)
*rpmG*-U_6_AU	0.2 ± 0.23 (89%)
*rpmG*-U_6_A	<0.5 (66%)^a^
rpmG-3′	1 ± 0.52 (89%)^b^
rpmG-U_5_	0.47 ± 0.27 (85%)
rpmG-U_4_	2.6 ± 3.5 (58%)
rpmG-U_2_	>50 (27%)
*rpmG*-noU	>50 (6%)
AniS-3′	5 ± 0.4 (91%)^b^
AniS-U_7_	0.82 ± 0.21 (88%)
AniS-U_6_	0.7 ± 0.62 (79%)
AniS-U_5_	6.5 ± 1.3 (90%)
AniS-U_4_	0.9 ± 0.58 (53%)
AniS-U_2_	>50 (14%)
AniS-noU	n.d.

The *K*_d_ values were obtained by fitting the data using the quadratic equation. The average *K*_d_ values with standard deviations were calculated from at least three independent experiments. (n.d.) The binding was essentially undetected up to 50 nM concentration of ProQ.

^a^The data could not be accurately fit, and the *K*_d_ range was estimated based on the data points distribution versus the protein concentration.

^b^Data from Table 1.

When the binding of RNAs derived from AniS-3′ was compared, the data showed that the derivatives with 3′ tails of six or seven uridines of length, AniS-U_6_ and AniS-U_7_, bound tightest to ProQ (Fig. 2C,D; Table 2; Supplemental Fig. S7B). The RNA with the longest, 8-uridine 3′ tail, AniS-3′, bound ProQ fivefold weaker than AniS-U_6_ and AniS-U_7_, but with similar affinity as the 3 nt shorter AniS-U_5_. Although AniS-U_4_ showed tighter binding affinity than AniS-3′, it had a decreased maximum fraction bound of ∼50%. The binding of the shortest AniS-3′ derivatives, which had the 3′ tail of two uridines or no tail, was very weak or not detected (Fig. 2C,D; Table 2; Supplemental Fig. S7B). The data analysis using global fitting for AniS-3′ derived molecules with 3′ tails of four to eight uridines confirmed these conclusions (Supplemental Table S1). Hence, for AniS-3′-derived RNAs, the tightest binding was observed with the 3′ tail of six or seven uridines of length, while RNAs with tails longer or shorter than that bound weaker to ProQ.

The observation that shortening of the 3′ tails of transcription terminators of *rmpG*-3′ and AniS-3′ below four uridines weakened RNA binding to ProQ is similar to previous observations that shortening of the 3′ tail length below four uridines markedly weakened RNA binding to the FinO domain of *E. coli* ProQ (Stein et al. 2020), and that the 3′ tail of three uridines caused RocR RNA to bind much weaker to RocC protein than the 3′ tail of five uridines (Kim et al. 2022). Interestingly, the 3′ tail length of 9 nt permits tight ProQ binding by *rmpG*-3′, while the tail length of 8 nt weakens the binding of AniS-3′ (Fig. 2; Table 2). Because the three uridines closest to the terminator of *rmpG*-3′ are involved in base-pairing, it effectively shortens the single-stranded length of the 3′ tail. Hence, the differences between *rmpG*-3′ and AniS-3′ RNAs regarding the length of 3′ tail that is optimal for tight binding to *N. meningitidis* ProQ could be the result of the different involvement of their 3′ tails in RNA structure.

### The RNA binding by *N. meningitidis* ProQ depends on the sequence at the 5′ side of terminator stem

**FIGURE 3.**
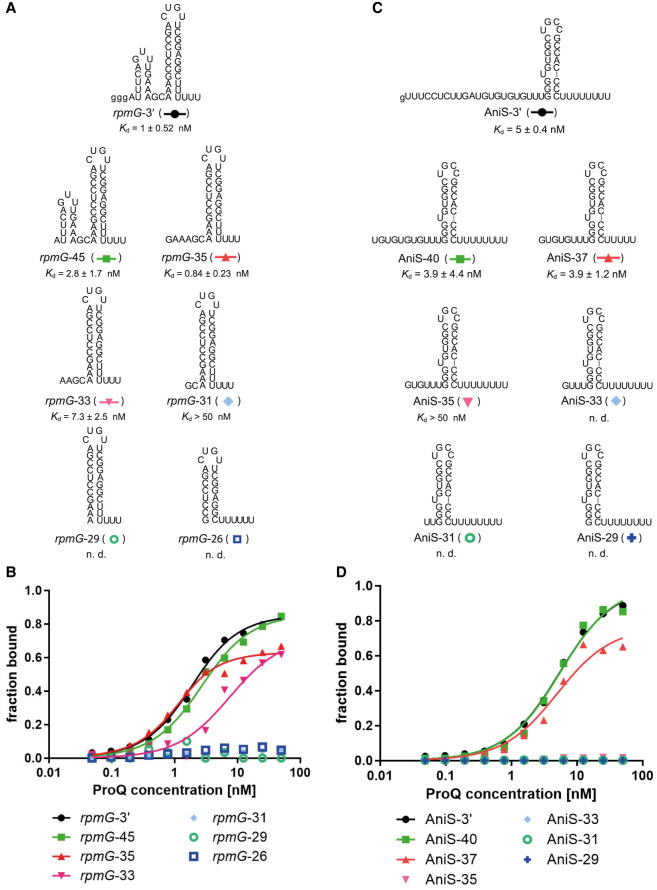
The 5′-terminal sequence preceding the terminator hairpin is involved in *rpmG*-3′ and AniS-3′ binding to the *N. meningitidis* ProQ protein. (*A*) *rpmG*-3′ constructs with different lengths of the 5′-terminal sequence. (*B*) The fitting of the ProQ binding data using the quadratic equation provided *K*_d_ values of 1.2 nM for *rpmG*-3′, 2.2 nM for *rpmG*-45, 0.6 nM for *rpmG*-35, and 7.0 nM for *rpmG*-33, while the binding for *rpmG*-31 did not reach saturation up to 50 nM concentration of the ProQ. The binding of *rpmG*-29 and *rpmG*-26 was essentially undetectable up to 50 nM concentration of the ProQ. (*C*) AniS-3′ constructs with different lengths of the 5′-terminal sequence. (*D*) The fitting of the ProQ binding data using the quadratic equation provided *K*_d_ values of 4.9 nM for AniS-3′, 5.0 nM for AniS-40, 4.5 nM for AniS-37, while the binding of AniS-35 was barely detected, and that of AniS-33, AniS-31, and AniS-29 was essentially undetected up to 50 nM concentration of the ProQ. The data in the plots for *rpmG*-3′ and AniS-3′ binding to ProQ are the same as in Figure 1. The lower case g denotes guanosine residue added on 5′ end to enable T7 RNA polymerase transcription. Gels corresponding to the data in the plots are shown in Supplemental Figure S8. The RNA secondary structure predictions were performed in the ViennaRNA program (Lorenz et al. 2011). The average equilibrium dissociation constant (*K*_d_) values and maximum RNA fraction bound calculated from at least three independent experiments are shown in Table 3.

In the next step, we analyzed how the length of the RNA sequence on the 5′ side of the terminator affects the binding of *rpmG*-3′ and AniS-3′ to ProQ (Fig. 3; Table 3; Supplemental Fig. S8). In these experiments, we used chemically synthesized oligoribonucleotides. The longest *rpmG*-3′-derived construct in these experiments was *rpmG*-45, which differed from *rpmG*-3′ by the absence of guanosine residues added to *rpmG*-3′ to enable efficient transcription (Fig. 3A). *rpmG*-45 bound ProQ somewhat weaker than *rpmG*-3′ but the binding achieved saturation at a similar maximum fraction bound. Then a 10 nt shorter construct was created, named *rpmG*-35. On the 5′ side of the G-C pair closing the terminator hairpin, this RNA had a 9 nt long sequence, which consisted of a 6 nt long single-stranded stretch and a 3 nt long stretch of adenosines base-paired with uridines of the 3′ tail. The affinity of *rpmG*-35 construct to ProQ had a similar *K*_d_ value as that of *rpmG*-45, which suggests that the 9 nt length of sequences consisting of single-stranded and double-stranded stretches on the 5′ side of the terminator is sufficient for tight ProQ binding. However, when the 5′ part of the molecule was truncated further 2 nt, the resulting *rpmG*-33 bound ProQ threefold weaker than *rpmG*-45. The difference between the ProQ binding affinities of *rpmG*-35 and *rpmG*-33 is also supported by the analysis using global data fitting (Supplemental Table S1). Further shortening resulted in *rpmG*-31, which had only two single-stranded residues on the 5′ end, and which bound ProQ very weakly. The binding of even more truncated *rpmG*-29 construct, which had the single-stranded portion of 5′ terminal RNA sequence completely removed, was also severely weakened as the binding to ProQ was not detected in the studied concentration range. Additionally, removing the 5′ terminal stretch of adenosine residues, which was base-paired with uridines of 3′ tail, resulted in a construct, named *rpmG*-26, which binding to ProQ was also not detected.

**TABLE 3.** The length of the sequence on the 5′ side of the Rho-independent terminator affects the binding of *rpmG*-3′ and AniS-3′ RNAs to *N. meningitidis* ProQ

^32^P-RNA	*K*_d_ [nM] (maximal fraction bound)
rpmG-3′	1 ± 0.52 (89%)^a^
rpmG-45	2.8 ± 1.7 (90%)
rpmG-35	0.84 ± 0.23 (71%)
rpmG-33	7.3 ± 2.5 (63%)
rpmG-31	>50 (8%)
rpmG-29	n.d.
rpmG-26	n.d.
AniS-3′	5 ± 0.4 (91%)^a^
AniS-40	3.9 ± 4.4 (83%)
AniS-37	3.9 ± 1.2 (60%)
AniS-35	>50 (2%)
AniS-33	n.d.
AniS-31	n.d.
AniS-29	n.d.

The *K*_d_ values were obtained by fitting the data using the quadratic equation. The average *K*_d_ values with standard deviations were calculated from at least three independent experiments. (n.d.) The binding was essentially undetected up to 50 nM concentration of ProQ.

^a^Data from Table 1.

When the 5′ truncated constructs of AniS-3′ were analyzed, we also observed dependence of ProQ binding affinity on the length of the 5′ terminal single-stranded sequence (Fig. 3C,D; Table 3; Supplemental Fig. S8; Supplemental Table S1). When the 5′ terminal single-stranded region was shortened to 11 nt in the AniS-40 construct it did not markedly affect the binding affinity as the *K*_d_ value was similar to that of AniS-3′. Of note, AniS-3′ does not contain an adenosine stretch bordering with the terminator. Hence, the whole sequence 5′ terminal to the G-C closing base pair of the terminator is single-stranded. Further truncation to 8 nt residues resulted in AniS-37, which had the same *K*_d_ value of ProQ binding, but the maximum fraction bound was lowered to ∼60%. Hence, the truncation of the 5′ terminal sequence to 8 nt did not markedly affect AniS-3′ binding to ProQ (Fig. 3C,D; Table 3; Supplemental Table S1). However, when the 5′ terminal single-stranded sequence was shortened to 6 nt in the AniS-35 construct, the binding to ProQ was abolished, and the same effect was observed when this sequence was further shortened to 4, and 2 nt or the whole sequence was removed, in AniS-33, AniS-31, and AniS-29, respectively (Fig. 3C,D; Table 3). Hence, for the efficient binding of AniS-3′ to ProQ, the length of at least 8 nt of single-stranded sequence on the 5′ side of the terminator is necessary. The importance of the single-stranded sequence on the 5′ side of the terminator for RNA binding to ProQ is consistent with a previous observation that a 7 nt long 5′ terminal single-stranded sequence was sufficient for tight binding of a model RNA construct derived from *cspE*-3′ to *E. coli* ProQ, while the complete removal of a 5′ terminal single-stranded sequence abolished the binding (Stein et al. 2023). In further support of the importance of the sequence 5′ of the terminator for *N. meningitidis* ProQ binding, it was previously shown that the CLIP-seq peak of AniS included also the sequence on the 5′ side of the terminator (Bauriedl et al. 2020). In summary, our data showed that a longer single-stranded sequence on the 5′ side was necessary for tight binding of AniS-3′ than *rpmG*-3′.

### *N. meningitidis* ProQ recognizes the lower part of the RNA terminator hairpin

**FIGURE 4.**
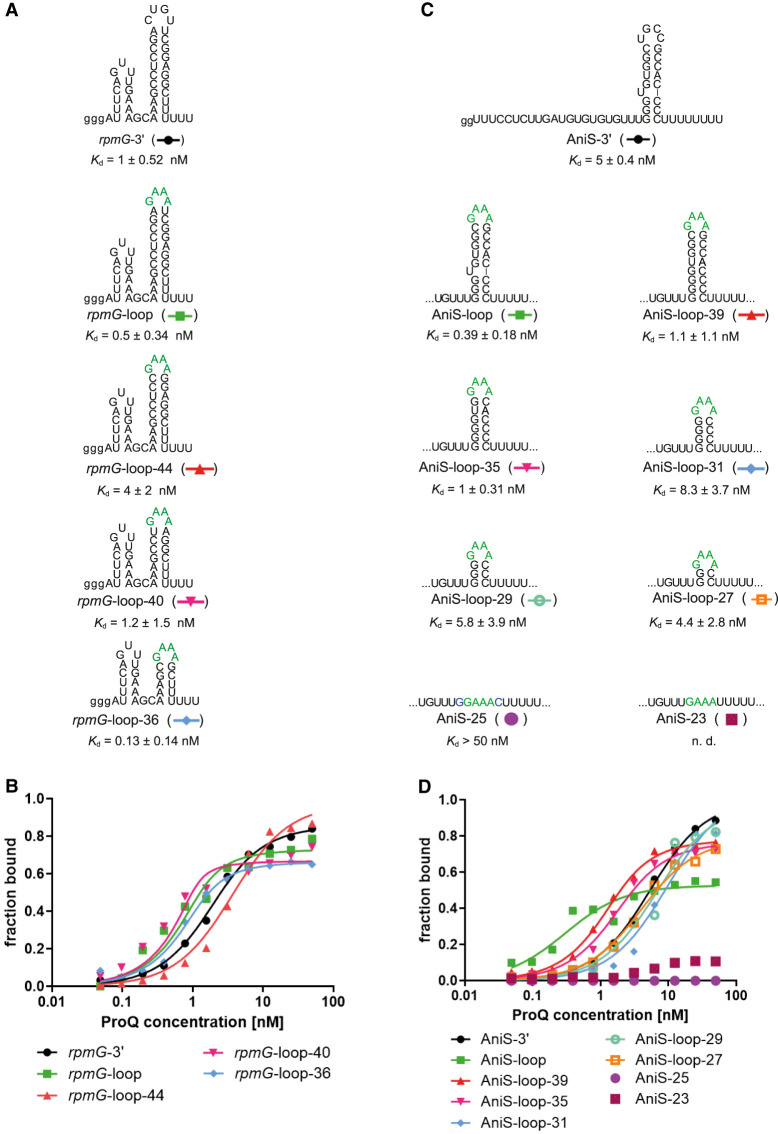
The lower part of the terminator hairpin is involved in *rpmG*-3′ and AniS-3′ binding to the *N. meningitidis* ProQ protein. (*A*) *rpmG*-3′ mutants with shorter terminator stems were constructed by replacement of the native apical loop CUGU with the tetraloop GAAA and gradual removal of base pairs from the top of the terminator stem. (*B*) The fitting of the ProQ binding data using the quadratic equation provided *K*_d_ values of 1.2 nM for *rpmG*-3′, 0.3 nM for *rpmG*-loop, 3.2 nM for *rpmG*-loop-44, 0.6 nM for *rpmG*-loop-40, and 0.3 nM for *rpmG*-loop-36. (*C*) AniS-3′ mutants with shorter terminator stems were constructed by replacement of the native apical loop UGCC with the tetraloop GAAA, removing the bulge, and gradual removal of base pairs from the top of the terminator stem. (*D*) The fitting of the ProQ binding data using the quadratic equation provided *K*_d_ values of 4.9 nM for AniS-3′, 0.7 nM for AniS-loop-39, 1.3 nM for AniS-loop-35, 9.2 nM for AniS-loop-31, 6.2 nM for AniS-loop-29, 3.5 nM for AniS-loop-27, while the binding for AniS-25 was barely detected, and AniS-23 was essentially undetected up to 50 nM concentration of the ProQ. The fitting of data for AniS-loop RNA to the equation for one site-specific binding with the Hill slope model provided *K*_d_ value of 0.1 nM. The data in the plots for *rpmG*-3′ and AniS-3′ binding to ProQ are the same as in Figure 1. The lower case g denotes guanosine residue added on 5′ end to enable T7 RNA polymerase transcription. Green font indicates GAAA tetraloop. Gels corresponding to the data in the plots are shown in Supplemental Figure S9. The RNA secondary structure predictions were performed in the *ViennaRNA* program (Lorenz et al. 2011). The average equilibrium dissociation constant (*K*_d_) values and maximum RNA fraction bound calculated from at least three independent experiments are shown in Table 4.

To test what is the contribution of the double-stranded portion of the terminator hairpin to RNA binding of ProQ, we designed variants of *rpmG*-3′ and AniS-3′, with gradually shortened terminator hairpin stems (Fig. 4; Table 4; Supplemental Fig. S9; Supplemental Table S1). At first, we replaced the apical loop of the terminator hairpin of *rpmG*-3′ with GAAA tetraloop to increase the stability of the shortened hairpin (*rpmG*-loop). Then, we gradually shortened the hairpin stem in 2 bp steps, thus creating *rpmG*-loop-44, *rpmG*-loop-40, and *rpmG*-loop-36 (Fig. 4A). We did not design a shorter construct, because it was predicted by *ViennaRNA* software not to retain a hairpin structure. The *rpmG*-loop-44 construct bound ProQ eightfold weaker than *rpmG*-loop, the *rpmG*-loop-40 construct bound ProQ with similar affinity as *rpmG*-loop, and the *rpmG*-loop-36 construct bound ProQ tighter than the *rpmG*-loop construct (Fig. 4A,B; Table 4; Supplemental Fig. S9A; Supplemental Table S1). The fact that even the construct with the shortest stem bound tightly to *N. meningitidis* ProQ suggests that ProQ binds the lower part of the terminator hairpin of *rpmG*-3′.

**TABLE 4.** The bottom part of the terminator stem of *rpmG*-3′ and AniS-3′ RNAs is recognized by *N. meningitidis* ProQ

^32^P-RNA	*K*_d_ [nM] (maximal fraction bound)
rpmG-3′	1 ± 0.52 (89%)^a^
*rpmG*-loop	0.5 ± 0.34 (81%)
*rpmG*-loop-44	4 ± 2 (91%)
*rpmG*-loop-40	1.2 ± 1.5 (80%)
*rpmG*-loop-36	0.13 ± 0.14 (64%)
AniS-3′	5 ± 0.4 (91%)^a^
AniS-loop	0.39 ± 0.18 (53%)
AniS-loop-39	1.1 ± 1.1 (72%)
AniS-loop-35	1 ± 0.31 (75%)
AniS-loop-31	8.3 ± 3.7 (82%)
AniS-loop-29	5.8 ± 3.9 (81%)
AniS-loop-27	4.4 ± 2.8 (62%)
AniS-25	>50% (9%)
AniS-23	n.d.

The *K*_d_ values were obtained by fitting the data using the quadratic equation, except for the data for the AniS-loop construct, which were fit using the equation for one site-specific binding with the Hill slope model. The average *K*_d_ values with standard deviations were calculated from at least three independent experiments. (n.d.) The binding was essentially undetected up to 50 nM concentration of ProQ.

^a^Data from Table 1.

Next, we designed the constructs of AniS-3′ sRNA with shortened terminator stems (Fig. 4C,D; Table 4; Supplemental Fig. S9B; Supplemental Table S1). In this series of molecules, the apical loop of AniS-3′ was also replaced with GAAA tetraloop. Additionally, we removed the single-uridine bulge located above the third base pair of the hairpin stem, thus creating a construct, which we named AniS-loop-39. Because this construct bound tightly to ProQ, and had a continuous double-stranded stem (Fig. 4C,D; Table 4; Supplemental Fig. S9B; Supplemental Table S1), we then designed truncated constructs based on AniS-loop-39. The data showed that shortening this construct by 2 bp to AniS-loop-35, which had a 6 bp stem, did not weaken the binding. On the other hand, further shortening in 2 bp increments to AniS-loop-31, AniS-loop-29, and AniS-loop-27, which had the shortest stem consisting of only two G-C base pairs, resulted in at least fourfold weaker binding in comparison to AniS-loop-39. However, even AniS-loop-27 bound tightly to ProQ with nanomolar *K*_d_. Complete removal of the hairpin stem in AniS-loop-25 and AniS-loop-23 fully abolished the binding. This suggests that the lowest two G-C base pairs are the part of the terminator hairpin of AniS-3′, which is essential for the binding to ProQ. The involvement of the lower parts of the terminator hairpins of *rpmG*-3′ and AniS-3′ in binding to ProQ is consistent with previous observations that this region is important for RNA binding by other FinO-domain proteins (Arthur et al. 2011; Stein et al. 2020; Kim et al. 2022).

### The 2′-OH and 3′-OH groups of the 3′ terminal ribose are important for RNA binding by *N. meningitidis* ProQ

**FIGURE 5.**
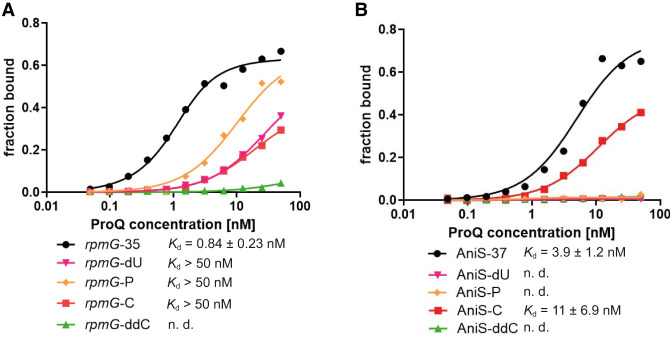
3′-terminal uridine is specifically recognized through the ribose 2′- and 3′-OH groups when RNA is bound by the *N. meningitidis* ProQ protein. (*A*) The fitting of the ProQ binding data using the quadratic equation provided *K*_d_ values of 0.6 nM for *rpmG*-35, while the binding of *rpmG*-dU, *rpmG*-P, and *rpmG*-C did not reach saturation up to 50 nM concentration of the ProQ. The binding of *rpmG*-ddC was essentially undetectable up to 50 nM concentration of the ProQ. (*B*) The fitting of the ProQ binding data using the quadratic equation provided *K*_d_ values of 4.5 nM for AniS-37 and 10.5 nM for AniS-C, while the binding for AniS-dU, AniS-P*,* and AniS-ddC was essentially undetectable up to 50 nM concentration of the ProQ. The data in the plots for *rpmG*-35 and AniS-37 binding to ProQ are the same as in Figure 3. Gels corresponding to the data in the plots are shown in Supplemental Figure S10. The average equilibrium dissociation constant (*K*_d_) values and maximum RNA fraction bound calculated from at least three independent experiments are shown in Table 5.

To better understand how the terminator hairpin is recognized by ProQ, we explored what is the contribution of the terminal residue of the RNA 3′ tail to its binding by ProQ (Fig. 5; Table 5; Supplemental Fig. S10). To achieve that we designed RNA constructs derived from *rpmG*-3′ and AniS-3′, in which the 3′ terminal residue was modified in a way that could affect hydrogen bonding interactions. The modified RNAs were chemically synthesized. As the model RNAs, we selected the constructs *rpmG*-35 and AniS-37 (Fig. 3A,C), because they are shorter than *rpmG*-3′ and AniS-3′, respectively, but retain the ability to bind tightly to ProQ (Table 3). Both these molecules have a uridine as the 3′ terminal residue. We designed two derivatives of each of these RNAs with modifications of the 3′ terminal uridine. One of them had a 2′-deoxyribose, and the other one had the 3′-OH group phosphorylated. Additionally, for both RNAs we designed a derivative, in which the 3′ terminal uridine was replaced with cytidine, and another one, in which it was replaced with 2′,3′-dideoxycytidine.

**TABLE 5.** The 2′ and 3′ hydroxyl groups of the 3′-terminal nucleoside are important for RNA recognition by *N. meningitidis* ProQ

^32^P-RNA	*K*_d_ [nM] (maximal fraction bound)
rpmG-35	0.84 ± 0.23 (71%)^a^
rpmG-dU	>50 (35%)
rpmG-P	>50 (37%)
rpmG-C	>50 (23%)
*rpmG*-ddC	n.d.
AniS-37	3.9 ± 1.2 (60%)^a^
AniS-dU	n.d.
AniS-P	n.d.
AniS-C	11 ± 6.9 (43%)
AniS-ddC	n.d.

The *K*_d_ values were obtained by fitting the data using the quadratic equation. The average *K*_d_ values with standard deviations were calculated from at least three independent experiments. (n.d.) The binding was essentially undetected up to 50 nM concentration of ProQ.

^a^Data from Table 3.

When the binding of 3′-terminally modified constructs of *rpmG*-35 was compared, the data showed that all modifications weakened RNA binding to ProQ (Fig. 5A; Table 5; Supplemental Fig. S10A). When the 3′-OH group of terminal uridine was blocked by phosphorylation, it caused more than 10-fold weaker binding of *rpmG*-P in comparison to unmodified *rpmG*-35. Similar negative effect on binding affinity was observed when the 2′-OH group was removed by replacing ribose with 2′ deoxyribose in *rpmG*-dU construct (Fig. 5A). A similar weakening of binding to ProQ was observed when the 3′ terminal uridine was replaced with cytidine in *rpmG*-C. On the other hand, removing both 2′-OH and 3′-OH groups of the terminal cytidine in *rpmG*-ddC completely abolished its binding to ProQ. The effects of modifications on the binding of AniS-37 derived constructs were even stronger, because the binding of the three constructs, AniS-dU, AniS-P, and AniS-ddC, in which the 2′-OH and/or 3′-OH groups were modified, could not be detected (Fig. 5B; Table 5; Supplemental Fig. S10B). On the other hand, substituting the 3′ terminal uridine with cytidine had only a small effect on binding (Table 5; Supplemental Table S1). The observation that 2′-OH and 3′-OH groups of the 3′ terminal ribose are important for RNA binding by *N. meningitidis* ProQ is consistent with previous studies on the effects of modifying these groups on RNA binding by F-like plasmid FinO protein and *L. pneumophila* RocC (Arthur et al. 2011; Kim et al. 2022). The co-crystal structure of *L. pneumophila* RocC explains these effects by showing that the 2′-OH and 3′-OH groups of RocR RNA are within the hydrogen bonding distance to conserved amino acids of RocC protein (Kim et al. 2022). On the other hand, no direct contacts were observed in the crystal structure between the uracil base of the 3′ terminal nucleoside and the RocC protein (Kim et al. 2022), which is consistent with the small effect of replacing uridine to cytidine in AniS-37 (Fig. 5B; Table 5; Supplemental Fig. S10B). Hence, we hypothesize that the negative effect of cytidine substitution in *rpmG*-C could be a result of changes in local base-pairing involving the cytidine rather than the disruption of specific contacts with *N. meningitidis* ProQ.

### The nucleotide composition of the sequence on the 5′ side of the terminator hairpin affects the binding of *rpmG*-3′ and AniS-3′ to ProQ

Because it was previously observed that RNAs bound by *E. coli* ProQ often had A-rich motifs on the 5′ side of the terminators (Stein et al. 2020), we used WebLogo software to compare the nucleotide content of the 10 nt long sequence on the 5′ side of the terminator in the top 40 previously identified RNA ligands of *N. meningitidis* ProQ, in which the CLIP-seq peak overlapped with intrinsic transcription terminators (Supplemental Fig. S6; Supplemental Table S2; Bauriedl et al. 2020). As controls, we also compared the nucleotide contents of the corresponding sequences in the top 40 3′ UTRs and sRNAs identified as ligands of *N. meningitidis* Hfq using RIP-seq (Heidrich et al. 2017), and in randomly selected 98 transcripts of *N. meningitidis* transcriptome. The analysis showed that there were no statistically significant differences between these three data sets, and in all of them, a short sequence on the 5′ side of the terminator was enriched in adenosines (Supplemental Fig. S6; Supplemental Table S2). This shows that the A-enrichment on the 5′ side of the terminator is a general feature of *N. meningitidis* transcriptome. However, AniS is an exception, because it has a U-rich sequence motif in this region (Supplemental Table S2).

**FIGURE 6.**
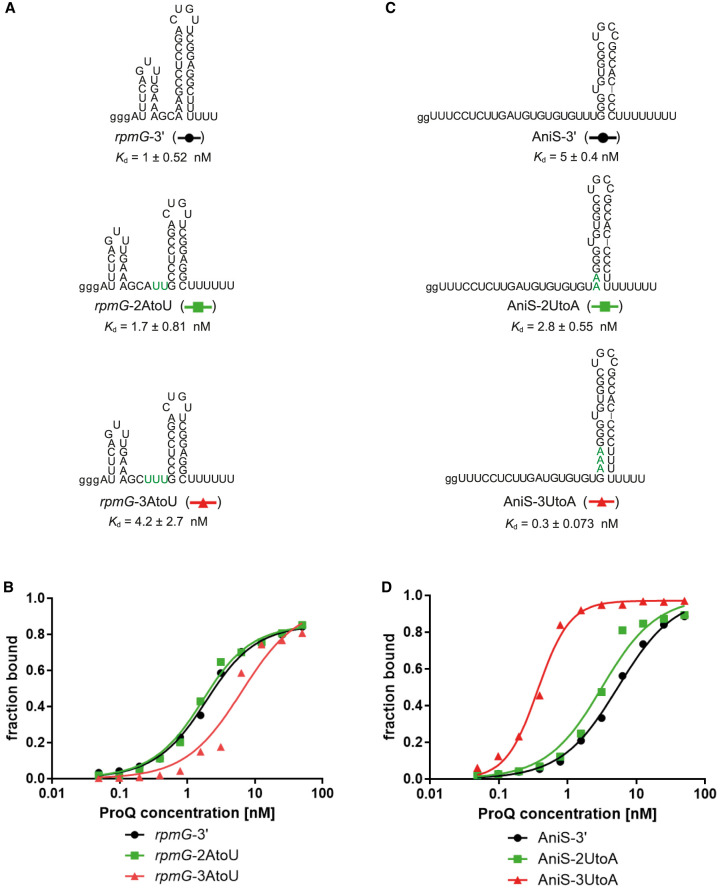
RNA mutations in the sequence at the 5′ side of the terminator stem affect the *rpmG*-3′ and AniS-3′ binding to *N. meningitidis* ProQ protein. (*A*) Secondary structures of *rpmG*-3′ and its mutants. (*B*) The fitting of the ProQ binding data using the quadratic equation provided *K*_d_ values of 1.2 nM for *rpmG*-3′, 1.1 nM for *rpmG*-2AtoU, and 5.8 nM for *rpmG*-3AtoU. (*C*) Secondary structures of AniS-3′ and its mutants. (*D*) The fitting of the ProQ binding data using the quadratic equation provided *K*_d_ values of 4.9 nM for AniS-3′ and 2.6 nM for AniS-2UtoA. The fitting of AniS-3UtoA to the equation for one site-specific binding with the Hill slope model provided a *K*_d_ value of 0.3 nM. The data in the plots for *rpmG*-3′ and AniS-3′ binding to ProQ are the same as in Figure 1. The lower case g denotes guanosine residue added on the 5′ end to enable T7 RNA polymerase transcription. Green font indicates the introduced substitutions. Gels corresponding to the data in the plots are shown in Supplemental Figure S11. The RNA secondary structure predictions were performed in the ViennaRNA program (Lorenz et al. 2011). The average equilibrium dissociation constant (*K*_d_) values and maximum RNA fraction bound calculated from at least three independent experiments are shown in Table 6.

To test if the nucleotide content of the sequence immediately 5′ of the terminator hairpin affects RNA binding to *N. meningitidis* ProQ, we introduced substitutions in this region in *rpmG*-3′ and AniS-3′ (Fig. 6; Table 6; Supplemental Fig. S11; Supplemental Table S1). While *rpmG*-3′ has a three-adenosine stretch opposite to its 3′ tail, AniS-3′ has a three uridine stretch in the corresponding position (Fig. 1A). To explore the importance of such a motif for ProQ binding, we designed two kinds of constructs. In *rpmG*-3′, we replaced either two or three adenosines of the A-rich motif by uridines, in this way creating *rpmG*-2AtoU and *rpmG*-3AtoU (Fig. 6A). On the other hand, in AniS-3′, we substituted either two or three uridines present on the 5′ side of the AniS-3′ terminator by adenosines, thus creating AniS-2UtoA and AniS-3UtoA constructs (Fig. 6C). The data showed that substituting adenosines with uridines moderately weakened the ProQ binding of *rpmG*-2AtoU and *rpmG*-3AtoU, because the *K*_d_ values were about two- or fourfold weaker, respectively, for each construct (Fig. 6B; Table 6; Supplemental Fig. S11A; Supplemental Table S1). On the other hand, the substitutions of uridines to adenosines in corresponding positions of AniS-3′ strengthened the binding of AniS-2UtoA and AniS-3UtoA, because the *K*_d_ values were either twofold or more than 10-fold tighter, respectively, for each construct (Fig. 6D; Table 6; Supplemental Fig. S11B; Supplemental Table S1). Hence, these data suggest that *N. meningitidis* ProQ is optimized to bind to intrinsic transcription terminators that have a stretch of A nucleotides on the 5′ side of the terminator hairpin, which are often involved in base-pairing with the uridine nucleotides of the 3′ tails.

**TABLE 6.** Mutations in the sequence on the 5′ side of the terminator stem affect the binding of the *rpmG*-3′ and AniS-3′ to *N. meningitidis* ProQ

^32^P-RNA	*K*_d_ [nM] (maximal fraction bound)
rpmG-3′	1 ± 0.52 (89%)^a^
*rpmG*-2AtoU	1.7 ± 0.81 (91%)
*rpmG*-3AtoU	4.2 ± 2.7 (78%)
AniS-3′	5 ± 0.4 (91%)^a^
AniS-2UtoA	2.8 ± 0.55 (88%)
AniS-3UtoA	0.3 ± 0.073 (97%)

The *K*_d_ values were obtained by fitting the data using the quadratic equation, except the data for AniS-3UtoA which was fit using the equation for one site-specific binding with the Hill slope model. The average *K*_d_ values with standard deviations were calculated from at least three independent experiments.

^a^Data from Table 1.

## DISCUSSION

Our results showed that the minimal ProQ protein from *N. meningitidis* recognizes three distinct parts of the intrinsic transcription terminator structures, which are the 5′ adjacent single-stranded sequence, the lower part of the terminator hairpin and the 3′ terminal tail (Figs. 2–4; Tables 2–4; Supplemental Figs. S7–S9). The same regions of the terminator are also important for RNA binding by other FinO-domain proteins including the F-like plasmid FinO protein, and the FinO domains of *E. coli* ProQ and *L. pneumophila* RocC proteins (Jerome and Frost 1999; Arthur et al. 2011; Stein et al. 2020, 2023; Kim et al. 2022).

The data presented here and previous studies indicate that the base of the terminator hairpin is important for RNA binding by the FinO-domain proteins (Fig. 4; Table 4; Supplemental Fig. S9; Arthur et al. 2011; Holmqvist et al. 2018; Stein et al. 2020; Kim et al. 2022). The results of our studies showed that the minimal length of the double-stranded stem of the terminator hairpin in AniS-3′ and *rpmG*-3′, which was sufficient for tight binding to *N. meningitidis* ProQ, was 2 G-C pairs, although in *rpmG*-3′ this minimal stem was additionally extended by three A-U pairs including the uridines of the 3′ tail (Fig. 4A,B; Table 4; Supplemental Fig. S9A). The essential role of the two lowest G-C base pairs was also shown for the binding of truncated *malM*-3′ mutants to the FinO domain of *E. coli* ProQ (Stein et al. 2020). Additionally, the role of the base of the terminator hairpin in FinP RNA binding to the FinO protein was shown using RNase footprinting (Arthur et al. 2011). The importance of the base of the terminator is also supported by the observation that the disruption of three C-G base pairs, including a closing base pair of the terminator stem of the 3′ UTR of *cspE* mRNA, abolished its binding by *S. enterica* ProQ (Holmqvist et al. 2018). Finally, the experiments with mutants of RocR RNA showed that shortening of its terminator stem to 5 bp did not weaken its binding to the FinO domain of *L. pneumophila* RocC (Kim et al. 2022). These observations are consistent with the crystal structure, which showed that several amino acid residues of the FinO domain of *L. pneumophila* RocC are within the hydrogen bonding distance to the lowest 5 bp of the terminator hairpin of RocR (Kim et al. 2022).

The single-stranded sequence on the 5′ side of the terminator has varied contributions to RNA binding by the FinO-domain proteins (Jerome and Frost 1999; Attaiech et al. 2016; Kim et al. 2022; Stein et al. 2023). The results presented here showed that truncation of the 5′ sequence to 5 nt or less in *rpmG*-3′ (including two single-stranded and three double-stranded nucleotides) or to six single-stranded nucleotides or less in AniS-3′ abolished their binding to *N. meningitidis* ProQ (Fig. 3; Table 3; Supplemental Fig. S8). The importance of the single-stranded sequence on the 5′ side of the transcription terminator was also reported for the binding of other FinO-domain proteins. It was previously observed that the binding of *E. coli* ProQ to a model RNA derived from *cspE*-3′ was abolished when the 5′ sequence was truncated to four single-stranded nucleotides or when it was completely removed (Stein et al. 2023). The strength of binding of the F-like plasmid FinO protein to a fragment of FinP RNA was dependent on a 4 nt long single-stranded sequence on the 5′ side of the terminator hairpin of FinP, and when this sequence was transferred on another hairpin, it also improved its binding by FinO (Jerome and Frost 1999). However, the complete removal of the 5′-adjacent sequence from the terminator of FinP only moderately affected the FinO binding (Jerome and Frost 1999). Additionally, a short 1 nt sequence on the 5′ side of the terminator was sufficient for the binding of a fragment of RocR RNA to the FinO domain of *L. pneumophila* RocC protein (Attaiech et al. 2016; Kim et al. 2022). These data suggest that RNA binding by both *N. meningitidis* ProQ and *E. coli* ProQ is strongly dependent on the single-stranded sequence on the 5′ side of the terminator, while this sequence could have smaller contributions to RNA binding by the F-like plasmid FinO and *L. pneumophila* RocC proteins.

There are subtle differences in the length of the 3′ tail, which is optimal for tight RNA binding by *N. meningitidis* ProQ and by other FinO-domain proteins (Jerome and Frost 1999; Stein et al. 2020; Kim et al. 2022). We observed that for *rpmG*-3′, the constructs with the tail length of 5–9 nt bound tightly to *N. meningitidis* ProQ, but shortening the tail below five uridines gradually weakened the binding (Fig. 2A,B; Table 2; Supplemental Fig. S7A). Additionally, extending the length of the 3′ tail of *rpmG*-3′ to 13 residues essentially abolished the binding (Supplemental Fig. S4). In contrast, for AniS-3′ we observed the tightest binding for the 3′ tail length of six or seven uridines, while elongating the tail to eight uridines weakened the binding, and also shortening the tail to less than four uridines abolished the binding (Fig. 2C,D; Table 2; Supplemental Fig. S7B). The presence of U-rich 3′ tails in the binding sites of *E. coli* ProQ and *S. enterica* ProQ was previously detected by CLIP-seq and RIL-seq studies (Holmqvist et al. 2018; Melamed et al. 2020). It was also shown that shortening of the 3′ tails of *malM*-3′ and *cspE*-3′ RNAs weakened their binding by *E. coli* ProQ (Stein et al. 2020). However, there were differences in the minimal tail lengths sufficient for *E. coli* ProQ binding by different RNAs, because for *malM*-3′, the 3′ tail length of four single-stranded uridines was sufficient for tight binding, while for *cspE*-3′ the 3′ tail length of six uridines, which included two single-stranded and four double-stranded residues, was necessary for tight binding (Stein et al. 2020). The essential importance of the 3′ tail was also shown for the binding of FinP RNA by the FinO protein, where truncating the 3′ tail of FinP from GAU_4_ to only GA essentially abolished the binding (Jerome and Frost 1999). Additionally, it was also observed using gel-shift assay that the binding affinities of FinO protein to RNAs derived from RocR, which had the 3′ tail lengths of three, five, or eight uridines, were quite similar (Kim et al. 2022). In contrast, the optimal tail length of RocR for binding to *L. pneumophila* RocC FinO domain was 5 nt, and either shortening or elongating it strongly decreased the binding (Kim et al. 2022). This preference for a specific length of the 3′ tail was explained by the crystal structure, which showed that the two terminal residues of the 3′ tail of RocR form hydrogen bonds with conserved residues of the FinO domain of RocC (Kim et al. 2022). The fact that the terminal residue of the 3′ tail has to be appropriately positioned for binding to conserved residues in the binding pocket of the FinO domain may be an important factor determining the optimal length of the tail, because either too short or too long 3′ tails would not be correctly positioned for these interactions. This suggests that the differences observed in the length of the 3′ tail optimal for tight RNA binding to different FinO-domain proteins could result from differences in the RNA-binding sites in different FinO domains or from differences in RNA sequence or structure, which could affect the positioning of the terminus of the 3′ tail in relation to the binding pocket in the FinO domains.

Our data showed that modifications of 2′-OH and 3′-OH groups of the 3′ terminal ribose are strongly detrimental for the binding of RNAs derived from *rpmG*-3′ and AniS-3′ to *N. meningitidis* ProQ (Fig. 5; Table 5; Supplemental Fig. S10). The removal of both 2′-OH and 3′-OH groups abolished the ProQ binding of *rpmG*-ddC and AniS-ddC mutants. The binding was also weakened by the removal of the 3′-OH group only or by blocking of the 2′-OH group by a phosphate (Fig. 5; Table 5; Supplemental Fig. S10). The 2′-OH and 3′-OH groups of the terminal nucleoside are also important for RNA binding by other FinO-domain proteins. The gel-shift-monitored RNA binding to the F-like plasmid FinO protein was abolished by the phosphorylation of the 3′-OH group, and strongly weakened by the blocking of both hydroxyl groups by 2′,3′-dialdehyde (Arthur et al. 2011). The phosphorylation of the 3′-OH group also abolished RocR RNA binding by *L. pneumophila* RocC, which was monitored using isothermal titration calorimetry (Kim et al. 2022). The reason for the importance of these hydroxyl groups in the binding has been explained by the crystal structure of *L. pneumophila* RocC, which showed that the 2′-OH and 3′-OH groups of the terminal uridine are within hydrogen bonding distance to peptide bond amino groups of conserved amino acid residues in type II β-turn between helices 3 and 4 of RocC (Kim et al. 2022). Interestingly, the contribution of the 3′-terminal 3′-OH group to RNA binding has been also observed for *Salmonella enterica* Hfq (Sauer and Weichenrieder 2011), which suggests that contacts with the hydroxyl groups of the 3′ terminal nucleoside are a common feature of bacterial proteins which recognize RNAs at their 3′ ends.

The substitution of an A-rich stretch on the 5′ side of the terminator of *rpmG*-3′ weakens its binding to *N. meningitidis* ProQ, while the substitution of a U-rich stretch in the corresponding position of AniS-3′ strengthens its binding to ProQ (Fig. 6; Table 6; Supplemental Fig. S11). We have previously observed that in RNA ligands of ProQ identified in *E. coli* and *S. enterica,* the sequence of the corresponding region is enriched in adenosine residues, as opposed to RNA ligands of Hfq, where this region is uridine-enriched (Holmqvist et al. 2018; Melamed et al. 2020; Stein et al. 2020). However, when we compared the corresponding sequence in RNAs bound by *N. meningitidis* ProQ (Bauriedl et al. 2020) and Hfq (Heidrich et al. 2017), we found that in both of these groups of RNAs, this sequence is A-enriched, and that the adenosine enrichment of this region is a general feature of *N. meningitidis* transcriptome (Supplemental Fig. S6; Supplemental Table S2). We note that because the identification of Hfq ligands in *N. meningitidis* was obtained using RIP-seq (Heidrich et al. 2017), it is not possible to distinguish whether the Hfq-binding sites were located at the terminator structures or elsewhere in the sequence. Interestingly, among 40 RNAs bound by Hfq, which were included in our sequence logo analysis, are sRNAs AniS, RcoF1, RcoF2, and 3′ UTR of NMV_1651, which have U-rich motifs in this region (Supplemental Table S2; Fantappie et al. 2011; Heidrich et al. 2017; Bauriedl et al. 2020). The fact that the substitution of uridines to adenosines in the region improved the ProQ binding to AniS, while the substitution of adenosines to uridines in the corresponding region weakened the binding of *rpmG*-3′ (Fig. 6; Table 6), supports the importance of the sequence 5′ adjacent to the terminator to RNA recognition by *N. meningitidis* ProQ.

In summary, the fact that *N. meningitidis* ProQ consists only of the FinO domain, but is capable of recognizing a subset of RNAs in this bacterium (Bauriedl et al. 2020) suggests that the interactions between a FinO domain and its binding site within an RNA molecule are sufficient to ensure specific RNA recognition. The data presented here show that the RNA sequence and structure elements that are recognized by *N. meningitidis* ProQ include the base of the intrinsic transcription terminator hairpin together with flanking sequences (Figs. 1–6). The role of the junction between the double-stranded stem and the surrounding single-stranded sequences in RNA recognition by the FinO domains is consistent with the recent crystal structure of a complex of *L. pneumophila* RocC with RocR RNA (Kim et al. 2022), which showed that the RNA binding pocket in the FinO domain of RocC binds the base of the terminator hairpin of RocR as well as the terminus of its 3′-tail. It is also supported by previous observations that mutations at the base of the intrinsic terminator hairpin and in the surrounding sequence affected RNA binding to mutants of the FinO domain of *E. coli* ProQ (Stein et al. 2023). While *N. meningitidis* ProQ, similarly as *E. coli* and *S. enterica* ProQ, is a global RNA-binding FinO-domain protein, there are also narrow RNA-binding specificity FinO-domain proteins, such as F-like plasmid FinO and *L. pneumophila* RocC. We hypothesize that interactions between a FinO domain and the intrinsic transcription terminator structure in its RNA ligand could be used distinctly by different FinO-domain proteins to ensure specific RNA recognition. Overall, our studies showed that the minimal ProQ from *N. meningitidis* recognizes RNAs in a generally similar way as the isolated FinO domains from other FinO-domain proteins. However, there are certain differences between them, which are related mainly to the sequence and length of single-stranded RNA sequences surrounding the terminator hairpin, which are required for optimal binding by *N. meningitidis* ProQ.

## MATERIALS AND METHODS

### Protein preparation

The sequence of *N. meningitidis* ProQ was cloned from pTYB11-ProQMenningo (a kind gift of Prof. Jörg Vogel, University of Würzburg) into expression vector pET-15b by PCR amplification and restriction digestion with BamH1 in *E. coli* DH5α. The resulting construct had an N-terminal cleavable His_6_-tag followed by a TEV protease recognition site (Supplemental Table S3). After cleavage, ProQ had a single additional serine residue on the N terminus. The expression plasmid was transformed into *E. coli* BL21 Δ*hfq* strain (a kind gift of Prof. Agnieszka Szalewska-Pałasz, University of Gdańsk). *N. meningitidis* ProQ was purified essentially as described previously for *E. coli* ProQ (Stein et al. 2020). In short, N-terminally His_6_-tagged ProQ was purified using nickel affinity chromatography, which was followed by heparin affinity chromatography to remove contaminating nucleic acids. After cleaving off the His_6_-tag using TEV protease, the tag was removed using the second nickel affinity chromatography, which was followed by size-exclusion chromatography. The purified ProQ was stored in a buffer consisting of 50 mM Tris, pH 7.5, 300 mM NaCl, 10% glycerol, and 1 mM EDTA in 10 µL aliquots of 10 μM concentration at −80°C. The aliquots were used without refreezing. The molecular weight of the purified protein with additional N-terminal serine residue remaining from the TEV cleavage site was determined by MALDI-TOF as 15,614.9 Da, which agrees with the calculated mass of 15,614.7 Da. The protein concentration was determined by measuring the absorption at 280 nm using an extinction coefficient of 5240 M^−1^ cm^−1^.

### RNA preparation

The DNA templates used for in vitro transcription were obtained by Taq polymerase extension of chemically synthesized overlapping oligodeoxyribonucleotides (Metabion) (Supplemental Table S4). RNA molecules were transcribed with T7 RNA polymerase and purified using denaturing gel electrophoresis, as described (Milligan et al. 1987; Olejniczak 2011). In the next step, RNAs were 5′-^32^P labeled using T4 polynucleotide kinase (Thermo Scientific), followed by phenol-chloroform extraction, purification using denaturing gel, and precipitation with ethanol. The obtained RNAs were dissolved in water and stored at −20°C. Chemically synthesized RNA oligos (Metabion) (Supplemental Table S5) were purified with denaturing gel electrophoresis followed by ^32^P-labeling.

### RNA binding assay

Before use, RNA molecules were denatured for 2 min at 90°C followed by 5 min refolding on ice. The concentration series of *N. meningitidis* ProQ was prepared by twofold dilutions from the concentration of 50 nM. In all binding reactions, 1 nM ^32^P-labeled RNA was mixed with the protein sample in binding buffer consisting of 25 mM Tris, pH 7.5, 150 mM NaCl, 5% glycerol, and 1 mM MgCl_2_, and incubated for 30 min at room temperature in low-protein binding microplates pretreated with 0.0025% bovine serum albumin solution. After incubation, reactions were loaded onto a 6% polyacrylamide gel (19:1) at 4°C. After the electrophoresis, gels were vacuum-dried and exposed to phosphor screens overnight. The signal was quantified using a phosphorimager (Fuji FLA-5000) and MultiGauge software, and the data were fit to the quadratic equation in GraphPad Prism software. The fitting of the binding data to obtain *K*_d_ values was performed for those reactions where the binding reached the maximum fraction bound of at least 40%. When the RNA-ProQ complex formed, but the maximum fraction bound was <40%, we assumed that the *K*_d_ value was higher than 50 nM, which was the highest ProQ concentration used. The average *K*_d_ values were calculated from at least three independent experiments.

## SUPPLEMENTAL MATERIAL

Supplemental material is available for this article.
